# Computational methods for protein localization prediction

**DOI:** 10.1016/j.csbj.2021.10.023

**Published:** 2021-10-19

**Authors:** Yuexu Jiang, Duolin Wang, Weiwei Wang, Dong Xu

**Affiliations:** Department of Electrical Engineering and Computer Science, Bond Life Sciences Center, University of Missouri, Columbia, MO, USA

**Keywords:** Protein localization prediction, Computational methods, Review

## Abstract

The accurate annotation of protein localization is crucial in understanding protein function in tandem with a broad range of applications such as pathological analysis and drug design. Since most proteins do not have experimentally-determined localization information, the computational prediction of protein localization has been an active research area for more than two decades. In particular, recent machine-learning advancements have fueled the development of new methods in protein localization prediction. In this review paper, we first categorize the main features and algorithms used for protein localization prediction. Then, we summarize a list of protein localization prediction tools in terms of their coverage, characteristics, and accessibility to help users find suitable tools based on their needs. Next, we evaluate some of these tools on a benchmark dataset. Finally, we provide an outlook on the future exploration of protein localization methods.

## 1 Introduction

Cells contain well-organized compartments with different protein constituents. Although most proteins are synthesized in the cytosol, about half of them are transported into or across at least one cellular membrane to reach their functional destination [1], [2], [3]. The aberrant localization of proteins usually has harmful effects, including diseases in humans and animals and poor traits in plants [4], [5], [6], [7]. Hence, studying the mechanism of protein localization is essential in a broad range of applications, such as plant breeding, pathological analysis, and the therapeutic modification of disease-related protein mislocalization [5], [8]. Protein localization is a complicated biological process controlled by many factors, such as signal peptides, protein trafficking, protein–protein interactions, folding, and alternative splicing [5], [9]. Among these, protein localization guided by targeting peptides is the most common mechanism [10] and includes pre-sequences and internal signals [11], [12]. Pre-sequences are found at the N- or C-terminus of protein sequences with enrichment of charged or hydrophobic amino acids, while internal signals are located in the middle of a sequence. How precursor proteins are directed to their target organelles is only partially understood [11], and only a small number of targeting peptides (particularly internal signals) have been experimentally identified. According to UniProt annotation (release 2020_05), out of the reviewed 20,394 human proteins, 7348 (36.0%) proteins have localization annotation with experimental verification, while only 3608 (17.7%) proteins have known targeting peptides. Furthermore, limited sub-organelle compartment localization data are available. According to a recent search that we conducted on 16,213 human proteins in ten human organelles, 5882 (36.3%) proteins had experimentally verified organellar localization annotation, while only 3518 (21.7%) proteins had experimentally verified sub-organellar localization annotation. Targeting peptide and sub-organelle data for non-human species are even sparser.

Several experimental methods can be used for protein localization analysis. Quantitative mass spectrometric readouts allow for the identification of proteins across fractions [13], [14], [15], [16]. Spatially and temporally resolved proteomic maps in living cells can be obtained by targetable peroxidase [17], [18], [19]. Techniques such as immunofluorescence and high-resolution confocal microscopy have enabled the visual estimation of protein localization within a single cell [20], [21], [22], [23], [24]. One problem with experimental methods is that their throughput is relatively low. In addition, experimental protein localization identification requires a great deal of time and resources. Importantly, experimental and computational protein localization identification approaches are complementary to each other. Experimental annotations are typically used as true labels for computational methods. Computational models are trained using these ground truth data to predict the localization of other proteins. Due to their cost-effective, automated, and high-throughput nature, computational methods are helpful for the large-scale characterization of protein subcellular locations.

**Fig. 1:**
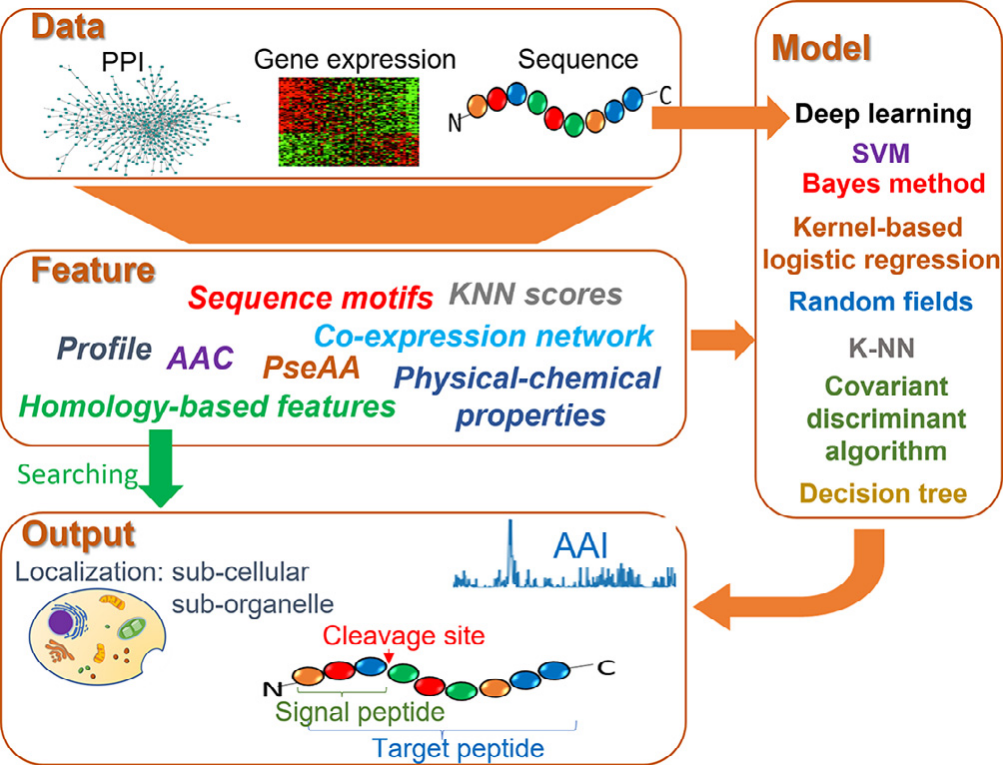
Relationships among the data, features, models, and prediction outputs in the computational prediction of protein localization. Sequence data can be converted into different features before feeding the data to a classifier model. Some classification models take raw data (e.g., one-hot-encoding of protein sequences for deep learning) as input, while others use engineered features. Localization prediction (at the sub-cellular and/or suborganellar level) is the most common output. Some methods also provide side product predictions such as target peptides, signal peptide cleavage sites, and mechanism interpretability at amino-acid-level resolution (AAI). Homology-based methods are special in the sense that they can make predictions directly based on homology-based features, such as the GO terms of homologous proteins.

Several papers have reviewed protein localization prediction methods. The review of [25] focuses on methods for bacterial protein localization prediction. Other reviews [26], [27] mainly cover protein sequence features (such as targeting peptides) in localization prediction. The methods reviewed in [28] predict protein function taxonomies, such as the Functional Catalogue, Enzyme Commission, or Gene Ontology, rather than specific cellular components. Another review mainly discusses web-based prediction tools for human protein subcellular localization [29]. General methods and tools for protein localization prediction are introduced in the reviews of [30], [31], [32], [33], which have a scope similar to ours. However, the most recent review in the literature [30], [31], [32] was published in 2014. Many new methods have been proposed since then that have greatly improved prediction accuracy, especially deep-learning methods. This review focuses on these new methods and tools in addition to previous representative methods. A less detailed review [33] was recently published. Compared to [33], this review separates the introduction of features, algorithms, and tools in greater detail so readers can better understand their relationships. Additionally, the applicability of the tools is considered, and only actively maintained tools are listed. Users can select the tools they need based on the information summarized and access them through the links provided. All the aforementioned features make this review unique and valuable. This review is organized as follows. In 2, 3, we analyze the features and classifiers that are often associated with different methods, respectively. Many of these methods provide standalone tools and/or web services that we summarize in Section 4. For each tool, information of target compartments, used algorithm, accessibility, etc. is given. In Section 5, a summary is provided together with promising directions for future protein localization prediction methods. The relationship of the data, features, and models used in computational protein localization prediction, as well as their outputs, are shown in Fig. 1. The features and main contributions of this review are summarized as follows:

• A systematic introduction of features, algorithms, methods, and tools, as well as their relationships related to protein localization.
• A comprehensive list of available protein localization prediction tools, many of which became available in recent years.
• Extensive evaluations of localization prediction tools/methods, providing insights on why some methods have better prediction performance than others.
• Significant discussion on the future direction of protein localization studies.

## 2 Data and features

### 2.1 Sequence-based features

Protein sequences are considered the most essential source of information for protein localization prediction, particularly terminal region sequences where targeting signals are likely to be found. Protein sequence information can be obtained from databases such as UniProt [34]. In addition, many types of features have been proposed based on protein sequences.

#### 2.1.1 Amino acid composition

The simplest feature representing a protein sequence is likely amino acid (AA) composition [35]. Given a protein sequence P, the AA composition of P can be expressed by

(1) $$P={\left[f_{1}f_{2}\cdots f_{20}\right]}^{T},$$

where $f_{u}\left(u=1,2,\cdots ,20\right)$ are the normalized occurrence frequencies of the 20 native amino acids in protein P.

#### 2.1.2 PseAA composition

The main shortcoming of using AA composition as a feature is its lack of protein sequence order information [31]. The concept of pseudo amino acid composition (PseAA) was proposed to address this problem [36] by representing a protein as a vector P:

(2) $$P={\left[p_{1}p_{2}\cdots p_{20}p_{20+1}\cdots p_{20+\lambda }\right]}^{T},(\lambda <L$$

where the 20+λ components are given by

(3) $$P_{u}=\left\{\begin{array}{cc} \frac{f_{u}}{\sum_{i=1}^{20}f_{i}+w\sum_{k=1}^{\lambda }\tau_{k}},\; & \left(1⩽u⩽20\right)\\ \frac{w\tau_{u-20}}{\sum_{i=1}^{20}f_{i}+w\sum_{k=1}^{\lambda }\tau_{k}}, & \left(20+1⩽u⩽20+\lambda \right) \end{array}\right)$$

where w is a weight factor set to 0.05 in the original paper [36], and $\tau_{k}$ is the k-th tier correlation factor, which reflects the sequence order correlation between all of the k-th most contiguous residues as formulated by

(4) $$\tau_{k}=\frac{1}{L-k}\sum_{i=1}^{L-k}J_{i,i+k},\;\left(K<L\right)$$

As in Eq. (2), the first 20 components are associated with the conventional amino acid composition of P, whereas the remaining components are the λ correlation factors that reflect the first tier, second tier, and so on up to the λ-th tier sequence order correlation patterns. These λfactors incorporate sequence order effects, and λ is a chosen hyperparameter (integer). The calculation of $\tau_{k}$ integrates the hydrophobicity values ($H_{1}$), hydrophilicity values ($H_{2}$), and side-chain masses (M) for amino acids *i* and *i + k* as

(5) $$J_{i,i+k}=\frac{1}{3}\left\{{\left[H_{1}\left(R_{i+k}\right)-H_{1}\left(R_{i}\right)\right]}^{2}+{\left[H_{2}\left(R_{i+k}\right)-H_{2}\left(R_{i}\right)\right]}^{2}+\right)\left({\left[M\left(R_{i+k}\right)-M\left(R_{i}\right)\right]}^{2}\right\}$$

Note that Eq. (5) is just one form for deriving the correlation factors. Other information, such as physicochemical distance and amphiphilic patterns, can also derive different types of PseAA composition.

#### 2.1.3 Homology information

As subcellular localization tends to be evolutionarily conserved [37], homology to a protein of known localization is often a good indicator of actual protein localization [38]. Such information can be derived via BLAST [39] or a more sensitive search method such as HHblits [40] against a database of proteins with known localization. One important source of known localization is the cellular component of Gene Ontology (GO) [41], which has been used to improve protein localization prediction performance [42], [43], [44], [45]. Homology information can also be obtained through protein structure similarity, as did in C-I-Tasser [46], a template-based method for protein structure and function prediction. In C-I-Tasser, the function prediction of a query protein is obtained by matching its structural model with proteins in the BioLiP function library via structure and sequence profile comparisons. Each entry in BioLiP contains GO terms so that the GO cellular localization of the query protein can be inferred.

#### 2.1.4 Evolutionary profiles

Evolutionary profiles, represented by Position-Specific Scoring Matrices (PSSMs), etc., provide informative input for protein localization prediction. PSSMs indicate the amino acid occurrence for each position in a protein multiple sequence alignment. PSSM scores are generally given as positive or negative values. A positive score means that the given amino acid substitution occurs more frequently in the alignment than expected by chance, while a negative score indicates that the substitution occurs less frequently than expected by chance. PSSMs can be created using PSI-BLAST, which finds similar protein sequences to a query sequence and then constructs a PSSM from the resulting alignment.

The BLOSUM (BLOcks SUbstitution Matrix) matrix [47] is a substitution matrix used for scoring alignments between evolutionarily divergent protein sequences. Several BLOSUM matrices exist using different alignment databases, which are named with sequence identity thresholds in the alignments. For example, BLOSUM62 is a matrix built using sequences with less than 62% similarity (sequences with ≥62% identity were clustered). BLOSUM62 is the default matrix for protein BLAST and is among the best for detecting weak protein similarities. Encoding with BLOSUM matrices is fast and provides a viable alternative if acquiring a PSSM is slow or unsuccessful [48], [49].

One particular usage of a sequence profile is as the profile kernel of an SVM. A key feature of the SVM optimization problem is that it depends only on the inner products of the feature vectors representing the input data. Several kernel functions have been proposed to avoid the explicit transformation of input data to feature vectors, explained as follows. Let Φ represent a mapping from the input space of protein sequences into a (possibly high-dimensional) vector space called the feature space. A string kernel is defined by $K\left(x,y\right)=\langle \Phi \left(x\right),\Phi \left(y\right)\rangle$, where x and y are sequences, e.g., $x=x_{1}x_{2}\cdots x_{N}$ from the alphabet Σ of amino acids ($\left|\Sigma \right|=20$, and the length $N=\left|x\right|$ depends on the sequence). Let $P\left(x\right)={\left\{p_{i}\left(a\right),a\in \Sigma \right\}}_{i=1}^{N}$ represent a profile for sequence x, with $p_{i}\left(a\right)$ denoting the emission probability of amino acid a in position i and $\sum_{a\in \Sigma }p_{i}\left(a\right)=1$ for each position i; a profile kernel is defined as $K\left(P(x),P(y)\right)$. The Fisher-SVM method [50] is a profile-kernel method that represents each protein sequence as a vector of Fisher scores extracted from a profile Hidden Markov Model (HMM) for a protein family. Kuang *et al.* proposed profile-based string kernels that use probabilistic profiles, such as those produced by the PSI-BLAST algorithm, to define position-dependent mutation neighborhoods along with protein sequences for inexact matching of k-length subsequences (“k-mers”) [51]. Such profile kernels are used in LocTree2 [52], an SVM-based method for protein localization prediction.

#### 2.1.5 Motifs

Certain sequence patterns may correlate with a specific subcellular localization due to localization signals or functional relationships [53]. This motif information can be retrieved from databases such as PROSITE [54] or by data mining. One special type of motifs represents targeting peptides, i.e., short sequences mainly present at protein termini that function like a postal code to specify an intracellular or extracellular destination [55]. Some methods predict the presence of targeting peptides as a side product in tandem with protein localization prediction [56], [57], while other methods use targeting peptides as input features to predict protein localization [53].

A sequence pattern can also be extracted through a sliding window of a k-mer sequence. The motif length *k* is often set based on specific needs or prior biological knowledge. For example, TetraMito [58] uses over-represented tetrapeptides (four continuous amino acids believed to encode a particular structure) as features to predict submitochondrial protein localization. A similar idea is used for sub-Golgi protein localization prediction by SubGolgi 2.0 [59], which uses an SVM classifier trained with g-gap dipeptide compositions (two amino acids with g residues between them). LOCALIZER [60] is another k-mer-based method for predicting plant and effector protein localization to chloroplasts, mitochondria, and nuclei. The motif length *k* varies in LOCALIZER to capture the target signals on protein sequences.

#### 2.1.6 Physical–chemical properties

As the name suggests, this feature uses AAs' physical and chemical properties to represent protein sequences. These previously calculated properties are stored in public databases. According to Venkatarajan and Braun [61], a comprehensive list of 237 physical–chemical properties of each amino acid was compiled from the SWISS-PROT [34] and dbGET [62] databases. They showed that the number of properties could be reduced while retaining approximately the same distribution of amino acids in the feature space. Notably, the correlation coefficient between the original and regenerated distances was more than 99% using the first five eigenvectors.

#### 2.1.7 Pre-train sequence embedding

Evolutionary information significantly benefits model prediction performance; however, as the number of proteins in databases increases, retrieving such information is often time-consuming. Additionally, evolutionary information is less powerful for small protein families, e.g., for proteins from the Dark Proteome [63]. One promising sequence embedding method uses the pre-train model adopted from Natural Language Processing (NLP). The pre-train model utilizes large, unlabeled text-corpora such as Wikipedia to conceptualize syntax and semantics. Pre-train methods such as Transformer [64], ELMo [65], Word2Vec [66], and Bert [67] employ self-learning and predict either the next word in a sentence given all previous words, the current word from a window of surrounding context words (or using the current word to predict the surrounding window of context words), or masked-out words given all unmasked words. Once trained, language models can extract features, referred to as embeddings, to use as input for subsequent supervised learning (transfer-learning). A similar strategy has been used for protein sequence embedding. SeqVec [68] uses ELMo on UniRef50 for pre-train embedding and transfer-learning for subcellular localization prediction. ProtTrans [69] employs different pre-training embedding models on UniRef and BFD data containing 2.1 billion protein sequences, which can also be used for protein localization prediction. In addition, a recent study showed that the pre-training embedding from language models followed by an attention-based deep-learning architecture could yield excellent performance in protein localization prediction even without using evolutionary information [70].

### 2.2 Protein interactions

If two proteins interact, they are neighbors of each other in a protein–protein-interaction (PPI) network. The localizations of the neighbors in a PPI network carry information about the localization of un-annotated proteins. For example, if the majority of a protein’s neighbors share the same localization, the protein is likely localized to the same location. The definition of protein interaction varies and can be based on physical connections or genetic regulations. Protein interaction data can be retrieved from databases such as MINT [71], DIP [72], BioGRID [73], and STRING [74].

### 2.3 Gene/protein expression

The rationale for using gene/protein expression as a feature is that genes/proteins in the same compartment at the organelle or suborganelle level tend to be co-expressed to perform related functions. Gene/protein expression information can be used in network form like the aforementioned protein interaction feature [75]. For example, an interaction is established if the expression correlation between two genes/proteins exceeds a predefined threshold. Gene/protein expression information can also be used to create features such as the k-nearest-neighbor (k-NN) scores in the MU-LOC method [76] or used as standalone features in the SLocX method [77]. Gene/protein expression data are widely available and can be downloaded from databases like the Gene Expression Omnibus (GEO) [78] and The Cancer Genome Atlas (TCGA) [79].

## 3 Classification algorithms

### 3.1 Support vector machine

Support vector machines (SVMs) [80] use kernel functions to map input vectors into high dimensional feature space and construct a hyperplane that maximizes the margin between different classes. SVMs can handle large feature spaces and effectively avoid overfitting.

The method proposed in [81] is an early SVM-based protein localization prediction approach. To deal with a multi-class classification problem, it uses AA composition as a feature to train SVM classifiers in a one-versus-rest fashion. pSLIP [82] employs the SVM method in conjunction with multiple physicochemical properties of amino acids to predict protein subcellular localization in eukaryotes across six different localizations. The Density-induced Support Vector Data Description (D-SVDD) is an extension of Conventional Support Vector Data Description (C-SVDD) that was introduced for a one-class classification task inspired by SVMs [83]. PLPD [84] uses AA-based and motif features to modify the D-SVDD for multi-class multi-label protein localization prediction, mainly from imbalanced training datasets. A two-level SVM system to predict protein localization is described in [85]. The first level consists of multiple SVMs using distinct AA-based features (AA composition and physical–chemical properties), and the SVM at the second level makes the final prediction. SLocX [77] uses an SVM to predict the subcellular localization of Arabidopsis proteins using gene expression and AA composition as features.

Recent SVM-based methods include SubMitoPred [86], which uses Pfam domain information to predict mitochondrial proteins and their sub-mitochondrial localization. ERPred [87] predicts ER-resident proteins by training an SVM with a combination of amino acid compositions from different parts of proteins. SubNucPred [88] predicts protein localization for 10 sub-nuclear locations sequentially by combining the presence or absence of a unique Pfam domain and an amino acid composition-based SVM model. CELLO2GO [89] combines an SVM-based localization prediction method with BLAST homology search. When homologous proteins with known localizations are available, their GO terms are used as possible functional annotations for a queried protein. Otherwise, the SVM classifier provides localization prediction. MultiP-SChlo [90] is another SVM-based method that predicts subchloroplast protein localization with multiple labels based on features such as PseAAC and AA properties. MKLoc [91] is an SVM-based method for multi-label protein localization prediction where protein sequences are represented by a 30-dimensional feature vector consisting of PseAAC, physical–chemical properties, motifs from PROSITE, and GO annotations. LocTree3 [42] improves upon LocTree2 [52] by including information about homologs, if available, through a PSI-BLAST search. MitoFates [92] is a prediction method for cleavable N-terminal mitochondrial targeting signals and their cleavage sites. Besides classical features such as AA composition, sequence profiles, and physical–chemical properties, MitoFates introduces novel sequence features, including positively charged amphiphilicity and presequence motifs, and trains an SVM classifier using these features. SChloro [93] converts a protein sequence into a PSSM profile and Kyte-Doolittle scale (average hydrophobicity). Two layers of SVMs are designed to predict targeting signal and membrane protein information. The final output predicts six sub-chloroplastic localizations by integrating the predictions from previous layers.

### 3.2 Probabilistic methods

#### 3.2.1 Bayes method

Probabilistic models, specifically Bayesian methods such as the Bayes Optimal Classifier or Bayesian Networks, make the most probable prediction for a new example. Bayesian methods use the Bayes Theorem [94] for calculating a conditional probability. They are also closely related to the Maximum a Posteriori (MAP), a probabilistic framework that finds the most probable hypothesis for a training dataset. In large real-world applications, the Bayes method usually assumes that different features are independent of each other, known as Naïve Bayes.

PSORT-B [53] and subsequent versions of it [95], [96] (with higher prediction coverage and refined subcategories), construct six analytical modules based on features including homology, motifs, and signal peptides. A query protein undergoes each of the six analyses and the results are combined using a Bayesian Network to generate a final probability value for each localization site.

#### 3.2.2 Kernel-based logistic regression

When determining the probability of a protein to be localized at a specific location given a PPI network, kernel-based logistic regression (KLR) considers the localization information of all the proteins in the network. The KLR model can be formulated as follows [97]. Given a protein–protein interaction network with N proteins $X_{1},\cdots ,X_{N}$, some of which have unknown localization, let

(6) $$X_{\left[-i\right]}=\left(X_{1},\cdots {,X}_{i-1},X_{i+1},\cdots ,X_{N}\right)$$

represent the protein set excluding protein *i*. Let

(7) $$M_{L}\left(i\right)=\sum_{j\neq i,x_{j}known}K\left(i,j\right)I\left\{x_{j}=L\right\}$$

represent the summed distances of protein *i* to proteins targeting localization *L*, where $K\left(i,j\right)$ is the kernel function for calculating the distances between two proteins in the network. Then, the KLR model is given by$\left(20+1⩽u⩽20+\lambda \right)$

(8) $$\log\frac{Pr(X_{i}=L|X_{\left[-i\right]},\theta )}{1-Pr(X_{i}=L|X_{\left[-i\right]},\theta )}=\gamma +\delta M_{!L}\left(i\right)+\eta M_{L}\left(i\right)$$

which means that the logit of $Pr(X_{i}=L|X_{\left[-i\right]},\theta )$, and the probability of protein *i* targeting location *L* is linear based on the summed distances of proteins targeting *L* or another location. Then, we have

(9) $$\mathrm{Pr}\left(X_{i}=L|X_{\left[-i\right]},\theta \right)=\frac{1}{1+e^{-(\gamma +\delta M_{!L}\left(i\right)+\eta M_{L}\left(i\right))}}$$

Note that the probability of being in each localization is calculated separately as a binary classification problem.

NetLoc [75] applies KLR to protein networks based on different relationships, including physical PPI, genetic PPI, and coexpression. In NetLoc, networks with high connectivity and a high percentage of interacting protein pairs targeting the same location lead to better prediction performance.

#### 3.2.3 Random Fields

Given a probability space, a random field *T(x)* defined in $R^{n}$ is a function such that for every fixed $x\in R^{n}$, *T(x)* is a random variable on the probability space [98]. Markov Random Fields (MRFs) and Conditional Random Fields (CRFs) have been used for protein localization prediction [56], [99]. An MRF of a graph G is a set of random variables corresponding to the nodes in G (random field) with a joint distribution that is Markov-constrained for G. In other words, the joint probability distribution associated with the MRF is subject to the Markov constraint given by G: for any two variables, $V_{i}$ and $V_{j}$, the value of $V_{i}$ is conditionally independent of $V_{j}$ given its neighbors $B_{i}$. In this case, the joint probability distribution $P(\left\{V_{i}\right\})$ factorizes according to G. In contrast, we can describe a CRF for a graph G as a set of random variables corresponding to the nodes in G, a subset ${\left\{X_{i}\right\}}_{i=1}^{n}$ of which are assumed always to be observed, and remaining variables ${\left\{Y_{i}\right\}}_{i=1}^{m}$ with a conditional distribution $P({\left\{Y_{i}\right\}}_{i=1}^{m}|{\left\{X_{i}\right\}}_{i=1}^{n})$ that is Markov-constrained for G. Both MRFs and CRFs typically fit a model that can be used for conditional inference in diverse settings. The main difference is that an MRF has no consistently designated “observed variables” and requires a joint distribution over all variables that adhere to the Markov constraints of G.

CRFs are used for signal peptide cleavage site prediction in DeepSig [99] and specific signal peptide prediction in SignalP 5.0 [56]. A tissue-specific subcellular localization prediction method is proposed in [100] using multi-label MRF. A tissue-specific network was constructed from generic physical PPI networks and tissue-specific functional associations, and tissue-specific localization annotations were obtained from HPA [101].

### 3.3 Distance-based methods

#### 3.3.1 k-nearest Neighbors (k-NN) classification

The k-NN algorithm is a nonparametric method used for classification and regression [102]. In both cases, the input consists of the k closest training examples in the data set. The output depends on whether the k-NN model is used for classification or regression. In k-NN classification, the output is class membership. An object is classified by a plurality vote of its neighbors, and assigned to the most common class of its k nearest neighbors (k is typically a small positive integer). If k = 1, then the object is simply assigned to the class of the single nearest neighbor.

WoLF PSORT [103] converts protein amino acid sequences into numerical localization features such as targeting signals, amino acid composition, and functional motifs. After conversion, a k-NN classifier is used for prediction. An idea similar to k-NN is used in [104], where a physical interaction network was obtained from BioGRID [73], and GO Cellular Component annotation was mapped onto the network, if available, for the corresponding protein (node). For a query protein, the percentage of its interactors associated with each target localization is calculated. The top two localizations are then reported as the prediction.

#### 3.3.2 Covariant discriminant algorithm based on Mahalanobis distance

The Mahalanobis distance [105] is a measure of the distance between a point P and a distribution D. It is essentially a multidimensional generalization to measure how many standard deviations away P is from the mean of D. This distance is zero if P is at the mean of D and grows as P moves away from the mean along each principal component axis. If each of these axes is re-scaled to have unit variance, then the Mahalanobis distance corresponds to the standard Euclidean distance in the transformed space. The Mahalanobis distance is thus unitless and scale-invariant and takes the correlations in a data set into account.

The Mahalanobis distance of an observation $\vec{x}={\left(x_{1},x_{2},x_{3},\cdots ,x_{N}\right)}^{T}$ from a set of observations with mean $\vec{\mu }={\left(\mu_{1},\mu_{2},\mu_{3},\cdots ,\mu_{N}\right)}^{T}$ and covariance matrix *S* is defined as:

(10) $$D_{M}\left(\vec{x}\right)=\sqrt{{\left(\vec{x}-\vec{\mu }\right)}^{T}S^{-1}\left(\vec{x}-\vec{\mu }\right)}$$

The similarity between standard vector ${\overset{¯}{X}}^{\xi }$ (normalized occurrence frequencies of the 20 AA from class ξ) and protein X is characterized by the covariant discriminant, as defined by Liu and Chou in [106]:

(11) $$F\left(X,{\overset{¯}{X}}^{\xi }\right)=D^{2}\left(X,{\overset{¯}{X}}^{\xi }\right)+ln\left(\lambda_{2}^{\xi }\lambda_{3}^{\xi }\lambda_{4}^{\xi }\cdots \lambda_{20}^{\xi }\right)$$

where the first term is the squared Mahalanobis distance, and $\lambda_{i}^{\xi }$ is the *i-*th eigenvalue of covariance matrix *S*.

The covariant discriminant algorithm is used in general protein localization prediction in [106], as well as in apoptosis protein localization prediction [107] and Golgi protein subtype prediction [108]. The features used in these methods are AA composition or Pseudo AAC.

### 3.4 Neural network/deep learning

An artificial neural network (ANN) is based on a collection of connected units or nodes called artificial neurons that loosely model the neurons in a biological brain. Each connection, like the synapses in a biological brain, can transmit a signal to other neurons. Each artificial neuron receives a signal and processes it, and the output of each neuron is computed by a non-linear function of the sum of its inputs. Increased GPU computing power and distributed computing allow the use of larger networks, which is known as “deep learning” [109]. Deep learning has become the hottest field in machine learning, and different architectures have been proposed, such as deep neural networks (DNNs) [110], convolutional neural networks (CNNs) [109], recurrent neural networks (RNNs) [111], [112], and attention mechanisms [113]. These deep learning methods, as well as traditional ANNs, have been applied in protein localization prediction. Due to the abstract feature extraction capability of deep learning models, artificial feature engineering is sometimes not required. Raw protein sequences can be given as inputs for many deep learning localization prediction methods [114], [115]. Among different deep learning architectures, RNNs are inherently suitable for processing protein sequences. Notably, a widely-adopted implementation of RNN, Long Short-Term Memory (LSTM), captures long-distance dependencies well [116]. LSTMs have been successfully applied in machine translation [117], [118], [119] and speech recognition [120], [121]. The methods used for these tasks can be applied to protein localization prediction by considering protein sequences as sentences and amino acids as words. CNNs are most commonly applied to analyze visual imagery [122]. A CNN uses shared-weight convolution kernels to slide along input features and provide feature maps for downstream calculations. The pooling operation reduces data dimension by combining the outputs of neuron clusters at one layer into a single neuron in the next layer. It is often desirable to apply CNNs to long protein sequences at the cost of losing single residue resolution for improved computational efficiency [48], [49], [123]. Moreover, CNN filters can be used to build position-weight matrices (PWMs) of sequence motifs, which can improve model interpretability [123]. The attention mechanism technique mimics cognitive attention [113] as it enhances the essential parts of input data and fades out the rest. This increases the signal-to-noise ratio and elucidates the contribution of features to the final prediction [48], [124], e.g., determines which amino acids are responsible for protein localization.

Several neural network/deep learning-based methods have been proposed for protein localization prediction. SCLpred [114] is an N-to-1 neural network for protein localization prediction capable of mapping a whole sequence into fixed-length properties so that no predefined feature is needed. A similar method was later used in SCLpred-EMS [125] to predict proteins in the endomembrane system and secretory pathway. DeepLoc [49] applies the CNN method, bidirectional LSTM [112], and the attention mechanism for predicting localization and detecting the regions in a protein sequence that contribute to localization prediction. The length of the embedding is the same as the input sequence, while the attention weight of each amino acid is a combination of several CNN filters of different receptive fields. This reduces the interpretation resolution of the model. The researchers also apply different embedding methods and illustrate that PSSM achieves significantly better performance than BLOSUM62 at the cost of increased computing time. MU-LOC [76] provides two models (SVM and DNN) to predict mitochondrial protein in plants. The features used include AA composition, PSSM, and gene expression. MULocDeep [48], developed from the same group that developed MU-LOC, is a recently developed deep learning method that extends target localization coverage to 10 main subcellular compartments and their suborganellar compartments with 44 localization classes in total. Its deep learning model consists of a bidirectional LSTM and a multi-head self-attention mechanism [124]. In addition to protein localization prediction, it sheds light on the mechanism of localization by highlighting regions on protein sequences as likely targeting peptides. DeepMito [126] is another deep learning method for sub-mitochondrial localization prediction using CNNs. Its features include physical–chemical properties and PSSM in addition to the one-hot encoding of raw sequences.

Some methods do not predict localization directly; rather, they predict the presence and location of targeting peptides from which the localization of corresponding proteins can be roughly inferred. For example, DeepSig [99] and SignalP 5.0 [56] predict signal peptides and their cleavage sites using deep-learning methods. DeepSig uses a CNN, while SignalP 5.0 applies a CNN, bidirectional LSTM, and a CRF for specific signal peptide prediction. TargetP 2.0 [57] is a deep learning model constructed by bidirectional LSTM and a multi-attention mechanism to predict N-terminal targeting signals that direct proteins to the secretory pathways, mitochondria, and chloroplasts, or other plastids. One attention head was assigned to each target class and trained as the second loss function to focus on the peptide cleavage site.

### 3.5 Decision tree-based methods

For prediction problems involving large-scale labeled data, neural networks tend to outperform other algorithms or frameworks. However, when it comes to small- to medium-sized data, decision tree-based algorithms are often considered optimal. A decision tree is a flowchart-like structure in which each internal node represents a “test” on an attribute, where each branch represents the outcome of the test, and each leaf node represents a class label. Decision tree-based methods have evolved over the years. For example, bagging (bootstrap aggregating) combines the predictions of multiple decision trees through a majority voting mechanism, random forests select only a subset of features at random to build a forest of decision trees, and boosting is achieved by sequentially minimizing the errors of previous models. Gradient boosting employs the gradient descent algorithm to minimize errors in sequential models. XGBoost [127] optimizes gradient boosting through parallel processing, tree-pruning, handling missing values, and regularization to avoid overfitting.

Decision-tree-based methods have also been applied to protein localization problems. Pang et al. developed a CNN-XGBoost model [128] to predict protein subcellular localization. A CNN acts as a feature extractor to automatically obtain features from a protein sequence, and an XGBoost classifier functions as a recognizer based on the output of the CNN. SubMito-XGBoost [129] extracts protein sequence-based features including g-gap dipeptide composition, PseAAC, and PSSM as feature vectors for boosting to predict protein submitochondrial localization. A similar study [130] extracts feature vectors of protein sequences using PSSM for a random forest model. Both [129], [130] apply the synthetic minority oversampling technique (SMOTE) to balance samples [131].

## 4 Tools

Many of the aforementioned methods mention web servers or standalone tools, but some of these are inaccessible due to lack of maintenance. We summarize a list of available protein localization prediction tools regarding their coverage, algorithms, accessibility, and other characteristics. These localization prediction tools (at the subcellular or suborganellar level) are shown in Table 1. Note that the BUSCA [132] and SubCons [133] tools are web servers that integrate different computational tools for protein subcellular localization prediction. The localization coverage of some tools, e.g., DeepSig and SignalP 2.0, is marked as SP (secretory pathway) in Table 1 because they are signal peptide prediction tools. Signal peptides direct proteins toward the secretory pathway, where the proteins are either located inside certain organelles (the endoplasmic reticulum, Golgi, or endosomes), secreted from the cell, or inserted into cellular membranes. Thus, the specific localization of these proteins is not unique. Some tools consider the secretory pathway as a low-resolution localization. For example, TargetP 2.0 predicts the presence of signal peptides and also predicts the targeting peptide for mitochondrial proteins and plastid proteins where unique protein localization can be inferred.

**Table 1:** Summary of protein localization prediction tools.

Tool	Cov_lv1	Cov_lv2	Species kingdom	Algorithm	Metrics	Year	Web server	Standalone
BUSCA [132]	1–4,7,11–14		Eu,Pro	Integrated method	F1, MCC	2018	http://busca.biocomp.unibo.it/	
CELLO2GO [89]	1–6,8–11,15		Eu,Pro,V	SVM and homology search	Acc	2014	http://cello.life.nctu.edu.tw/cello2go/	
MULocDeep [48]	1–10	1–10	Eu	LSTM + attention	Acc, MCC, Rec, Prec, ROC_AUC, P&R_AUC	2021	http://mu-loc.org/	√
DeepLoc [49]	1–10		Eu	CNN + LSTM + attention	Acc, MCC, Gorodkin measure	2017	https://services.healthtech.dtu.dk/service.php?DeepLoc-1.0	
TargetP 2.0 [57]	SP,4,7		Eu,Pro	LSTM + attention	Prec, Rec, F1, MCC	2019	https://services.healthtech.dtu.dk/service.php?TargetP-2.0	
MU-LOC [76]	4		P	SVM and neural network	Acc, Prec, F1, MCC	2018	http://136.32.161.178/	√
LocTree3 [42]	1–4,6–11		Eu,Pro	SVM and homology search	Acc, Std	2014	https://rostlab.org/services/loctree3/	
MitoFates [92]	4		Eu	SVM	Prec, Rec, MCC, ROC_AUC	2015	http://mitf.cbrc.jp/MitoFates/cgi-bin/top.cgi	√
LOCALIZER [60]	1,4,7		P	SVM	SN, SP, PPV, MCC, Acc	2017	http://localizer.csiro.au/	√
SignalP 5.0 [56]	SP		Eu,Pro	CNN, bidirectional LSTM, and CRF	MCC, Rec, Prec	2019	http://www.cbs.dtu.dk/services/SignalP/	√
DeepSig [99]	SP		Eu,Bac	CNN and CRF	MCC, FPR, F1	2018	https://deepsig.biocomp.unibo.it/welcome/default/index	√
PSORTb 3.0 [96]	2,3,14–16		Bac	SVM and homology search	Prec, Rec, Acc, MCC	2010	https://www.psort.org/psortb/	√
WoLF PSORT [103]	1–4,7,11		Eu	k-NN classifier	Acc	2007	https://wolfpsort.hgc.jp/	
SubCons [133]	1–4,6,8–11		Hum	Integrated method	F1, MCC	2017	https://subcons.bioinfo.se/	
TPpred 3.0 [136]	4,7		Eu	Integrated method	MCC, Prec, Rec	2015	https://tppred3.biocomp.unibo.it/tppred3	√
MultiLoc2 [44]	1–4,6–11		Eu	SVM	SN, SP, MCC	2009	https://abi-services.informatik.uni-tuebingen.de/multiloc2/webloc.cgi	√
YLoc [45]	1–4,6–11		Eu	Naïve Bayes and entropy-based discretization	F1, Acc	2010	https://abi-services.informatik.uni-tuebingen.de/yloc/webloc.cgi	√
SCLpred-EMS	SP		Eu	Neural network	SP, SN, FPR, MCC	2020	http://distilldeep.ucd.ie/SCLpred2/	
ERPred [87]	6		Eu	SVM	Acc, SN, SP, MCC	2017	http://proteininformatics.org/mkumar/erpred/index.html	√
SeqVec[68]	1–10		Eu	Language Model + FNN	Acc, MCC, FPR	2019	https://embed.protein.properties/	√
ProtTrans [69]	1–10		Eu	Language Model + FNN	Acc	2020	https://embed.protein.properties/	√
LA [70]	1–10		Eu	Language Model + attention	Acc	2021	https://embed.protein.properties/	√
DeepMito [126]		4	Eu	CNN	MCC, GCC	2019	http://busca.biocomp.unibo.it/deepmito/	√
SubGolgi v2 [59]	8	8	Eu	SVM	SN, Acc, MCC	2013	http://lin-group.cn/server/subGolgi2	
TetraMito [58]		4	Eu	SVM	SN, Acc, MCC	2013	http://lin-group.cn/server/TetraMito	
Schloro [93]		7	P	SVM	Acc, Rec, Prec, F1, ROC_AUC, MCC	2017	https://schloro.biocomp.unibo.it/welcome/default/index	√
SubMitoPred [86]	4	4	Eu	SVM	Acc	2017	http://proteininformatics.org/mkumar/submitopred/	√
SubNucPred [88]		1	Eu	SVM	Acc, SN, SP, MCC	2014	http://proteininformatics.org/mkumar/subnucpred/index.html	√

The localization coverage codes are: 1. nucleus; 2. cytoplasm; 3. extracellular; 4. mitochondrion; 5. cell membrane; 6. endoplasmic reticulum; 7. plastid/chloroplast; 8. Golgi apparatus; 9. lysosome/vacuole; 10. peroxisome; 11. plasma membrane; 12. organelle membrane; 13. endomembrane system; 14. outer membrane; 15. periplasmic; 16. cell wall; SP. secretory pathway.

Cov_lv1 represents subcellular localization coverage, and Cov_lv2 indicates that suborganellar localization predictions are provided for the organelle.

The species kingdom codes are: Eu (Eukaryota, including animal, plant, and fungi); Pro (Prokaryota, including Bacteria and Archaea); V (Virus); P (Plant); Bac (Bacteria); Hum (Human).

The metrics codes are: MCC (Matthews correlation coefficient), Acc (accuracy), SN (sensitivity), SP (specificity), Prec (precision), Rec (recall), ROC_AUC (area under receiver operating characteristic curve), P&R_AUC (area under precision & recall curve), GCC (Generalized Correlation Coefficient), PPV (positive predictive value), FPR (false positive rate).

To assess prediction tools, competitions can provide large-scale blind tests for objective evaluation. A well-known example is the CASP [134] in the protein structure prediction field. For protein localization prediction, the Critical Assessment of protein Function Annotation algorithms (CAFA) [135] is a good platform for such a purpose. CAFA requires a method to provide prediction in the form of cellular component ontology (CCO) terms. However, most methods reviewed in this paper predict UniProt's localization annotations rather than the CCO terms, and hence may not be assessed at CAFA directly. DeepLoc is a state-of-the-art method, and their dataset is often used by new methods for training and testing, as well as method comparison. Here, we used the DeepLoc dataset as a benchmark to evaluate some of the tools. The DeepLoc dataset was extracted from the UniProt database, release 2016_04. The protein dataset was filtered using the following criteria: eukaryotic, complete protein, encoded in the nucleus, longer than 40 amino acids, and experimentally verified (ECO:0000269) single localization annotation. Similar locations or subclasses of the same location were mapped to 10 main locations to increase the number of proteins per compartment (refer to Table 1 in [49] for details regarding the class distribution). A total of 13,858 proteins were obtained after the filtering process. PSI-CD-HIT [137] was used to cluster proteins with 30% identity or a 10^−6^ E-value cutoff, and the alignment was required to cover 80% of the shorter sequences, resulting in 8410 clusters for the whole dataset. The five-fold datasets generated had approximately the same number of proteins at each location. Four of the datasets were used for training and validation, and one was held out for testing. In this way, the redundancy between the training and testing datasets was reduced.

**Fig. 2:**
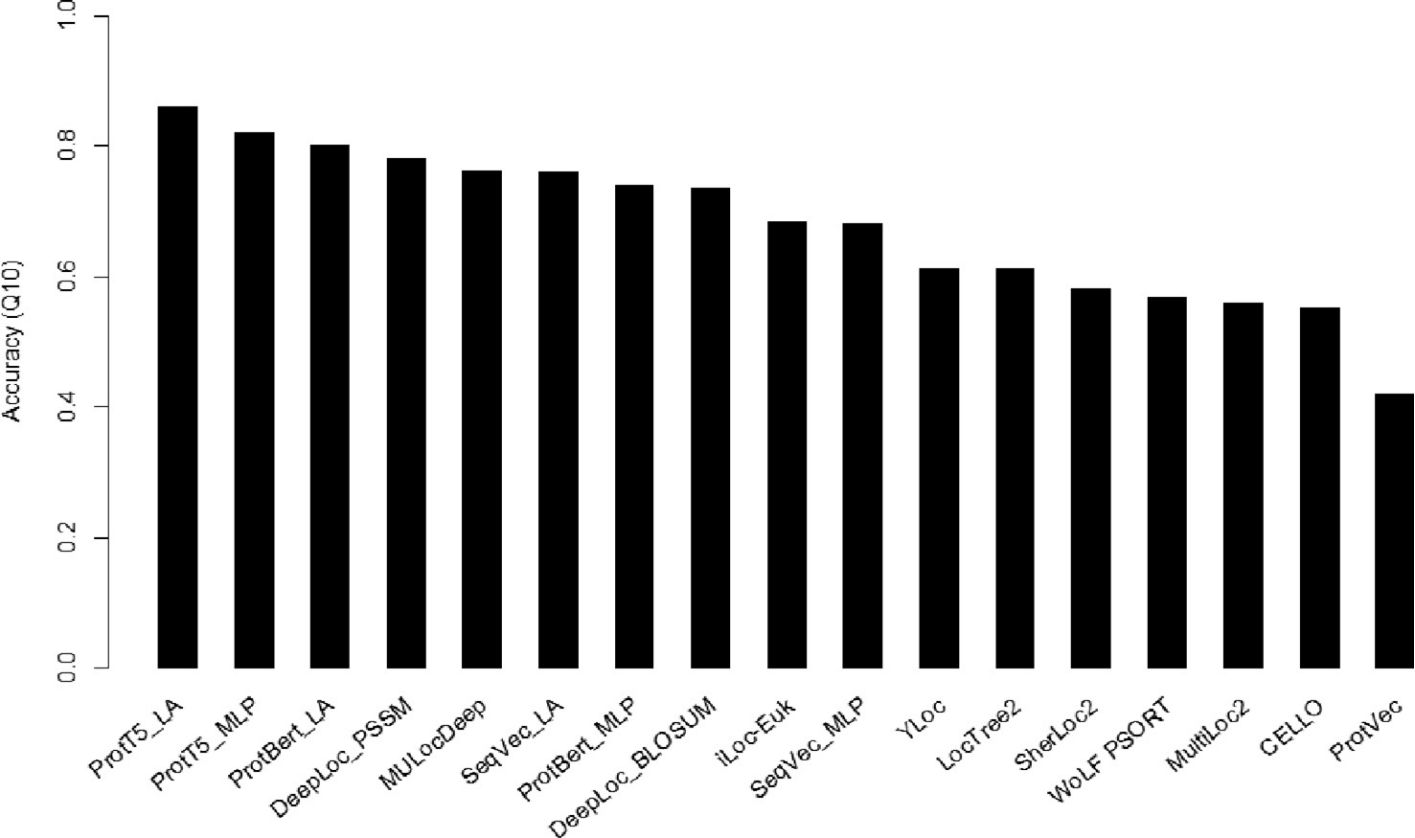
Evaluations of protein localization methods/tools. The criterion is the overall prediction accuracy for 10 main localizations. DeepLoc_PSSM and DeepLoc_BLOSUM are DeepLoc methods with PSSM and BLOSUM62 embedding, respectively. ProtT5_MLP and ProtBert_MLP are simple feed-forward neural networks in the ProtTrans method but using pre-train embeddings by T5 and Bert, respectively. ProtT5_LA and ProtBert_LA use the same two pre-trained models as above but are followed by an attention-based neural network.

The DeepLoc, MULocDeep, SeqVec, ProtVec, and ProtTrans methods were stringently trained and tested using the training and testing samples in the DeepLoc dataset. LocTree2, MultiLoc2, CELLO, WoLF PSORT, YLoc, SherLoc2, and iLoc-Euk were run on the testing samples in the DeepLoc dataset. Thus, their performance is potentially overestimated because redundancy control was not performed. All the evaluated methods could be applied to proteins in eukaryotic cells. In the cases where a method predicted more than ten locations, the predicted locations were mapped onto the ten locations in the DeepLoc dataset. Overall accuracy is used as the evaluation criterion. The evaluation performance is directly cited from [48], [49], [68], [69], [70]. As shown in Fig. 2, the deep learning-based methods (DeepLoc, MULocDeep, ProtTrans, and SeqVec) have overall better performance than the other methods, except for ProtVec [138], which uses Word2Vec, a context-independent embedding method. DeepLoc_PSSM achieves better performance than DeepLoc_BLOSUM, indicating that evolutionary information enhances localization prediction. By comparing the performance of pre-trained methods (ProtTrans and SeqVec) with other deep learning methods (DeepLoc and MULocDeep), we find that a simple deep learning architecture with pre-train embedding can achieve competitive or even better performance than delicately designed deep-learning models using evolutionary profile features.

## 5 Discussion and outlook

The computational prediction of protein localization has significantly improved prediction accuracy and localization mechanism studies over past two decades, especially with deep learning. However, the current methods still have limitations. For example, an 80% overall prediction accuracy shown in Fig. 2 does not mean that the localization prediction problem is 80% solved. In particular, many suborganellar localizations do not have sufficient data to build reliable prediction models. In this section, we discuss several areas for future exploration of localization analysis methods.

Protein localization problems have several biological characteristics. Many proteins can localize to more than one compartment. Some proteins are tissue-/cell type-specific, meaning their localization varies between different tissues or cell types. Proteins expressed at the correct location but with altered efficiency or concentration can also lead to illness. Thus, quantitively measuring or predicting protein localization in different tissues or cell types are in great demand. Additionally, proteins may be mislocalized due to mutations, which may have disease consequences [5]. Predicting mislocalization due to mutations is also challenging because it requires more sensitive methods with individual residue resolution.

Researchers could also pay more attention to biological interpretability when designing future localization analysis models. The mechanism of protein localization is complicated. In addition to targeting peptides, which are considered in some existing methods, other phenomena can affect/control protein localization. The trafficking machinery in cells controls the transport of molecules across membranes of organelles. Dysregulation of the protein trafficking machinery can have dramatic effects on general protein transport processes [139]. For example, the homozygous mutation R391H in the nucleoporin NUP155 has been shown to reduce nuclear envelope permeability and affect the export of Hsp70 mRNA and import of HSP70 protein [140]. Another fairly common method that affects protein localization involves binding partners that carry bound proteins between compartments. This mechanism allows for indirect control of protein localization by regulating the localization and concentration levels of binding partners, similar to the role of import receptors [9]. However, the prediction of protein localization changes affected by other proteins has not been explored. Furthermore, some localization signals are not contained within the linear peptide sequence of a cargo protein but are formed by the arrangement of amino acid residues on its surface. One advantage of such an arrangement is that conformational changes induced by allosteric events can disrupt or reform the localization signal transiently in response to the state of the protein [9]. Making protein localization analysis methods interpretable would allow us to answer “how” besides “where” a protein localizes, which has implications in pathology and drug design. The corresponding training data for such methods is currently lacking but may become available in the near future.

## CRediT authorship contribution statement

**Yuexu Jiang:** Conceptualization, Investigation, Visualization, Validation, Writing - original draft. **Duolin Wang:** Visualization, Writing - review & editing. **Weiwei Wang:** Validation. **Dong Xu:** Writing - review & editing, Supervision, Project administration, Funding acquisition.

## Declaration of Competing Interest

The authors declare that they have no known competing financial interests or personal relationships that could have appeared to influence the work reported in this paper.
